# Comparative effectiveness of rivaroxaban versus warfarin or dabigatran for the treatment of patients with non-valvular atrial fibrillation

**DOI:** 10.1186/s12872-017-0672-5

**Published:** 2017-09-06

**Authors:** Faye L. Norby, Lindsay G.S. Bengtson, Pamela L. Lutsey, Lin Y. Chen, Richard F. MacLehose, Alanna M. Chamberlain, Ian Rapson, Alvaro Alonso

**Affiliations:** 1 Division of Epidemiology and Community Health, School of Public Health, University of Minnesota, 1300 S 2nd St, Suite 300, Minneapolis, MN 55454 USA; 2 Health Economics and Outcomes Research, Life Sciences, Optum, Eden Prairie, MN USA; 3 Cardiac Arrhythmia Center, Cardiovascular Division, Department of Medicine, University of Minnesota Medical School, Minneapolis, MN USA; 4 Department of Health Sciences Research, Mayo Clinic, Rochester, MN USA; 5 Department of Epidemiology, Rollins School of Public Health, Emory University, Atlanta, GA USA

**Keywords:** Non-valvular atrial fibrillation, Stroke, Warfarin, Dabigatran, Rivaroxaban

## Abstract

**Background:**

Rivaroxaban is an oral anticoagulant approved in the US for prevention of stroke and systemic embolism in patients with non-valvular atrial fibrillation (NVAF). We determined the effectiveness and associated risks of rivaroxaban versus other oral anticoagulants in a large real-world population.

**Methods:**

We selected NVAF patients initiating oral anticoagulant use in 2010–2014 enrolled in MarketScan databases. Rivaroxaban users were matched with warfarin and dabigatran users by age, sex, enrolment date, anticoagulant initiation date, and high-dimensional propensity score. Study endpoints, including ischemic stroke, intracranial bleeding (ICB), myocardial infarction (MI), and gastrointestinal (GI) bleeding, were identified from inpatient diagnostic codes. Multivariable Cox models were used to assess associations between type of anticoagulant and outcomes.

**Results:**

The analysis included 44,340 rivaroxaban users matched to 89,400 warfarin and 16,957 dabigatran users (38% female, mean age 70) with 12 months of mean follow-up. Anticoagulant-naïve rivaroxaban initiators, but not those switching from warfarin, had lower risk of ischemic stroke [hazard ratio (HR) (95% confidence interval (CI)): 0.75 (0.62, 0.91)] and ICB [HR (95%CI): 0.55, (0.39, 0.78)] than warfarin users. In contrast, anticoagulant-experienced rivaroxaban initiators had higher risk of GI bleeding than warfarin users [HR (95%CI): 1.55 (1.32, 1.83)]. Endpoint rates were similar when comparing anticoagulant-naïve rivaroxaban and dabigatran initiators, with the exception of higher GI bleeding risk in rivaroxaban users [HR (95%CI) 1.28 (1.06, 1.54)]. There were no significant differences in the risk of MI among the comparison groups.

**Conclusion:**

In this large real-world sample of NVAF patients, effectiveness and risks of rivaroxaban versus warfarin differed by prior anticoagulant status, while effectiveness of rivaroxaban versus dabigatran differed in GI bleeding risk.

**Electronic supplementary material:**

The online version of this article (10.1186/s12872-017-0672-5) contains supplementary material, which is available to authorized users.

## Background

Atrial fibrillation (AF) is the most common sustained cardiac arrhythmia, with a lifetime risk of 1 in 4 in the general population, and an increasing prevalence as the population ages [1]. The estimated prevalence of AF in the United States (US) is expected to rise to 12.1 million by 2030 [2, 3]. Patients with any type of AF, whether permanent, persistent, or paroxysmal, and whether they are symptomatic or asymptomatic, are at increased risk of thromboembolic ischemic stroke, [4–7] with nonvalvular AF (NVAF) associated with a 5 times greater risk compared to those without NVAF [4].

For patients with diagnosed NVAF, the current ACC/AHA/HRS Guideline for the Management of Patients with AF recommends oral anticoagulation in those with a prior stroke or transient ischemic attack, or those with a moderate or greater risk of stroke (CHA_2_DS_2_-VASc score ≥ 1 in males or ≥2 in females) [8]. Vitamin K antagonist anticoagulants, with warfarin being the most common in the US, have been prescribed since the 1950’s as an oral anticoagulant for stroke prevention in patients with NVAF. Over the last six years, several non-vitamin K antagonist oral anticoagulants (NOACs) have been approved by the US Food and Drug Administration (FDA) to reduce the risk of stroke and systemic embolism in patients with NVAF.

Rivaroxaban is a direct factor Xa inhibitor approved by the FDA in November, 2011 for the prevention of stroke and systemic embolism in patients with NVAF. Rivaroxaban is administered as a single daily dose of 20 mg for most patients or 15 mg for those with reduced kidney function. A dose of 10 mg may be prescribed for the prevention or treatment of deep venous thrombosis (approved in July 2011). In the large randomized controlled trial, ROCKET AF, NVAF patients randomized to rivaroxaban experienced lower rates of stroke, intracranial bleeds, and fatal bleeding than those assigned to warfarin [9]. However, concerns were raised regarding international normalized ratio (INR) measures in the warfarin (control) arm of the clinical trial data [10, 11]. Published real-world studies of rivaroxaban vs. warfarin in NVAF patients report similar results as the clinical trials, but have focused on limited outcomes and have not stratified on patient characteristics [12–14].

Dabigatran, a direct thrombin inhibitor, was the first NOAC to be FDA-approved (October, 2010) for the prevention of stroke and systemic embolism in patients with NVAF. Data indicate dabigatran is associated with a lower risk of stroke and intracranial bleeds compared to warfarin, however, dabigatran users may be more at risk of GI bleeds and myocardial infarctions (MI) compared to warfarin users [15, 16]. Head-to-head comparisons of the effectiveness of dabigatran vs. rivaroxaban in NVAF patients indicate rivaroxaban initiators have an increased risk of intracranial bleeding and major bleeding, including GI bleeds [12, 17].

In this real-world study, we determined the effectiveness and associated risks of rivaroxaban vs. warfarin and rivaroxaban vs. dabigatran use in anticoagulant-naïve NVAF patients. We also assessed the effectiveness of rivaroxaban in patients who switched from warfarin compared to those who use only warfarin.

## Methods

### Study population

A retrospective cohort study was conducted using healthcare claims data from January 1st, 2010 through December 31st, 2014 from the Truven Health MarketScan® Commercial Claims and Encounters Database and the Medicare Supplemental and Coordination of Benefits Database (Truven Health Analytics, Inc., Ann Arbor, MI) [18]. The MarketScan Commercial Database includes health insurance claims spanning all levels of care, as well as enrolment data from large employers and health plans across the United States, providing private healthcare coverage for employees, their spouses, and dependents. The MarketScan Medicare Supplemental Database includes claims from individuals and their dependents with employer-sponsored Medicare Supplemental plans. Both databases link medical and outpatient prescription drug claims and encounter data with patient enrolment data to provide individual-specific clinical utilization, expenditure, and outcomes information across inpatient and outpatient services and outpatient pharmacy services. Patients with AF enrolled in the MarketScan Medicare Supplemental Database have similar demographic characteristics to patients with AF in the general fee-for-service Medicare population [19, 20].

The initial sample included 1,021,079 patients age 22–99 with at least one inpatient or 2 outpatient claims for AF 7 to 365 days apart (International Classification of Diseases, Ninth Revision, Clinical Modification (ICD-9-CM) codes 427.3, 427.31, and 427.32 in any position). We excluded patients with ICD-9-CM codes for valvular disease or procedure codes for valvular repair or replacement because the NOACs have received FDA approval for NVAF only (*n* = 92,098). The analytic sample was 522,620 once we restricted to individuals with at least one prescription for warfarin, rivaroxaban or dabigatran after their first AF claim between January 1, 2010 and December 31st, 2014. The final available sample was 227,799 after requiring ≥90 days of continuous enrolment prior to the first oral anticoagulant prescription. If a patient discontinued enrolment and then re-enrolled, we analysed their first period of enrolment. A systematic review of studies using ICD-9-CM codes for AF identification reported a positive predicted value (PPV) of approximately 90% and a sensitivity of approximately 80% [21]. All patient information is Health Insurance Portability and Accountability Act compliant, de-identified, commercially available secondary data, and therefore the Institutional Review Board at the University of Minnesota deemed this analysis exempt from review.

### Anticoagulant use and initial matching

Prescriptions for warfarin, rivaroxaban and dabigatran were identified following the first code for AF from 2010 to the end of 2014. Patients were initially categorized according to their first anticoagulant prescription during this period as a new warfarin user, a new rivaroxaban-only user, or a new dabigatran-only user. Warfarin users switching to rivaroxaban during follow-up were identified as switchers. Due to limited numbers, patients switching from dabigatran to rivaroxaban were not considered in this study, and neither were those taking other NOACS.

Each rivaroxaban-only initiator was matched with up to 3 warfarin-only initiators by age (± 3 years), sex, time since database enrolment (± 90 days) and drug initiation date (± 90 days). Computerized matching using a greedy matching algorithm was used to match rivaroxaban users to warfarin users [22]. New rivaroxaban users were matched 1:1 with new dabigatran users using the same matching criteria. Individuals switching from warfarin to rivaroxaban were matched with up to 5 warfarin-only users by age (± 3 years), sex, time since database enrolment (± 90 days) and warfarin initiation date (± 90 days). The date the individual switched to rivaroxaban (the index date) then became the index date for the matched warfarin-only user. Warfarin users must have had ≥90 days of warfarin use before the index date to be considered as a match. The validity of warfarin claims in administrative data has a PPV of 99% and a sensitivity of 94% [23]. Baseline characteristics after initial matching are provided in Additional file 1: Table S1.

### Outcome ascertainment

The main outcomes of interest included ischemic stroke, intracranial bleeding, MI, and GI bleeding, and were identified from inpatient claims using validated algorithms described below. In addition, 3 control outcomes of hip/pelvic fracture, breast/prostate cancer, and asthma were also obtained from inpatient claims. Hip/pelvic fracture was included a priori as a control outcome where no association with anticoagulant type was expected. A similar risk of hip/pelvic fracture by anticoagulant type would provide indirect evidence of no confounding. The control outcomes of breast/prostate cancer and asthma were added post-hoc after unexpected associations were observed between hip/pelvic fracture and anticoagulant type. Codes for these variables are listed in the Additional file 1: Tables S1-S8.

Ischemic stroke was defined based on the presence of ICD-9-CM codes 434.xx (occlusion of cerebral arteries) and 436.xx (acute but ill-defined cerebrovascular disease) as the primary discharge diagnosis in any inpatient claim following the index date. A PPV of >80% has been reported in several validation studies that used this definition [24]. Intracranial bleeding was defined based on the presence of ICD-9-CM codes 430 (subarachnoid haemorrhage) and 431 (intracerebral haemorrhage) as the primary discharge diagnosis in an inpatient claim following the index date. The PPV has been reported as >90% in many different validation studies [24]. MI was defined as the presence of ICD-9-CM codes 410.xx in the 1st or 2nd position of an inpatient discharge diagnosis. This excluded code 410.×2, which is used to indicate follow-up of the initial episode. The PPV for this algorithm is between 88 and 94% in validation studies [25, 26]. GI bleeding was defined by an algorithm developed by Cunningham et al. [27] that considers presence of bleeding-related ICD-9-CM codes in inpatient claims as primary and secondary diagnoses, presence of transfusion codes, and presence/absence of trauma codes to exclude trauma-related bleeding. The PPV of this algorithm is 86%, which is comparable to other peer-reviewed algorithms [26]. Hip/pelvic fracture, breast/prostate cancer and asthma were defined according to algorithms developed by the Centers for Medicare and Medicaid Chronic Condition Data Warehouse [28].

### Assessment of covariates

Pre-determined covariates were defined based on inpatient, outpatient and pharmacy claims that took place prior to the index date using validated published algorithms [27, 29]. Demographic characteristics, comorbidities, procedures and pharmacy prescription fills were ascertained. Comorbidities of interest were ascertained with published algorithms from inpatient and outpatient claims and include prior stroke/transient ischemic attack, haemorrhagic stroke, heart failure, MI, hypertension, diabetes, peripheral arterial disease, liver disease, kidney disease, chronic pulmonary disease, malignancies (except malignant skin neoplasm), metastatic cancer, history of bleeding, haematological disorders (anaemia, coagulation defects), dementia, depression, and alcohol abuse [27, 29]. Cardiac, vascular, gastrointestinal, and neurologic procedures also were identified from inpatient and outpatient claims. Presence of prescription fills for the following medication groups were ascertained: digoxin, clopidogrel, other antiplatelets, angiotensin-converting enzyme inhibitors, angiotension receptor blockers, ß-blockers, calcium channel blockers, antiarrhythmics, statins, and antidiabetic medications. The CHA_2_DS_2_-VASc score [30] was calculated at index date to determine stroke risk. Codes for these variables are listed in the Additional file 1: Tables S1-S8.

### Statistical analysis

High-dimensional propensity scores (HDPS) were calculated using methodology proposed by Schneeweiss et al., [31] and included the following pre-defined variables: age, age ≥ 75, calendar year, sex, the CHA_2_DS_2_-VASc score, any prevalent outcome before the start of the index date, and covariates listed above, which were obtained from inpatient and outpatient diagnostic codes and procedure codes, and outpatient pharmacy claims. HDPS were calculated with SAS macros developed by Rassen et al. and included the covariates described above and the most prioritized empirical covariates [32]. To define the empirical covariates, the data were categorized into 5 domains: inpatient diagnostic codes, inpatient procedure codes, outpatient diagnostic codes, outpatient procedure codes, and medications. Within each of the 5 domains, we selected the 200 most prevalent conditions. This resulted in 1000 covariates. All the covariates in the dimensions listed above were empirically rank ordered based on their potential for controlling confounding (i.e. strength of the covariate-outcome association and prevalence of the covariate) [33]. We selected the top 500 covariates based on this ordering. The 500 empirically-derived covariates, along with the pre-specified covariates mentioned above (also listed in Table 1), were included as covariates in a regression model to calculate the probability of receiving a DOAC versus warfarin. Separate HDPS were calculated for each of the anticoagulant comparison-outcome pairs (7 outcomes × 3 comparison groups = 21 total HDPS).

**Table 1:** Characteristics of atrial fibrillation patients by anticoagulant use, MarketScan, 2010–2014

	New Users		Switchers		New Users	
	New Rivaroxaban (*n* = 32,495)	Matched Warfarin (*n* = 45,496)	Rivaroxaban Switcher (*n* = 11,845)	Matched Warfarin (*n* = 43,904)	New Rivaroxaban (*n* = 16,957)	Matched Dabigatran (*n* = 16,957)
Age, years	69.3 ± 12.2	71.1 ± 12.5	71.2 ± 12.1	71.4 ± 12.0	67.2 ± 12.1	67.2 ± 12.1
Age ≥ 75 years	37.1	43.5	44.3	44.9	29.9	29.8
Female, %	38.7	40.1	39.3	39.5	34.1	34.2
Comorbidities, %						
Hypertension	66.0	62.8	84.8	82.5	61.9	59.2
Diabetes	25.7	26.7	35.3	35.4	23.4	23.5
Myocardial infarction	7.1	8.1	11.1	11.0	5.5	5.2
Heart failure	23.1	26.0	38.7	38.3	19.5	19.3
Ischemic stroke/TIA	15.5	17.2	29.1	27.0	12.1	12.2
Hemorrhagic stroke	0.6	0.8	1.7	1.6	0.3	0.3
PAD	12.2	14.1	23.7	23.1	8.8	8.5
Dementia	1.1	1.5	2.9	2.6	0.6	0.7
Renal Disease	7.6	10.3	14.1	15.5	5.3	5.0
Chronic pulmonary disease	21.4	22.5	34.6	32.7	17.8	17.1
Liver disease	3.6	3.6	7.0	6.6	2.9	2.9
Malignancy	10.9	11.3	17.1	16.5	8.5	7.8
Depression	7.0	7.5	13.7	11.9	5.5	5.6
Hematological disorders	7.6	9.7	22.5	21.8	5.6	5.1
Metastatic cancer	1.6	1.9	2.6	2.5	1.0	0.9
Alcohol abuse	0.4	0.4	0.6	0.6	0.3	0.3
GI bleed	4.4	4.9	12.1	11.5	3.4	3.2
Other bleed	2.4	3.0	8.3	8.0	1.7	1.5
CHA_2_DS_2_-VASC score	3.0 ± 1.9	3.2 ± 2.0	4.0 ± 2.1	3.9 ± 2.1	2.6 ± 1.8	2.6 ± 1.8
CHA_2_DS_2_-VASC score ≥ 2	75.5	79.5	87.4	86.8	69.4	68.5
Prior procedures, %						
Cardiac	54.6	53.6	81.1	79.4	52.2	50.5
Vascular	4.5	6.3	9.7	9.2	2.8	2.7
Gastrointestinal	21.1	20.8	42.3	39.6	17.5	16.6
Neurological	12.7	12.0	23.7	19.7	9.7	8.5
Medications, %						
Digoxin	11.4	13.3	23.0	22.8	10.9	11.2
Clopidogrel	9.5	9.6	11.0	10.5	7.9	7.3
Antiplatelets	1.8	1.8	2.1	1.9	1.4	1.2
Angiotensin-converting enzyme inhibitors	30.0	30.7	41.7	41.4	27.5	28.2
Angiotensin receptor blockers	20.2	19.2	26.5	24.9	18.8	17.7
Beta-blockers	63.9	61.8	77.3	75.6	61.5	60.8
Calcium channel blockers	36.0	34.9	46.5	44.1	33.6	32.9
Anti-arrhythmias	20.2	17.9	32.9	28.2	21.9	21.5
Statins	46.6	47.4	62.2	62.0	42.7	42.5
Diabetes medications	19.6	20.7	25.4	25.4	18.2	19.2
Initial dose of anticoagulant, %						
Rivaroxaban						
10 mg	6.2	--	5.4	--	4.8	
15 mg	18.2	--	21.0	--	15.2	
20 mg	75.6	--	73.7	--	80.0	
Warfarin						
< 5 mg	--	33.2	30.2	31.8	--	--
5 mg	--	48.0	51.6	49.3	--	--
> 5 mg	--	18.9	18.3	18.9	--	--
Dabigatran						
75 mg	--	--	--	--	--	11.9
150 mg	--	--	--	--	--	88.1

Values correspond to mean ± standard deviation or percentage

Separate models were used to compare 1) new rivaroxaban users to new warfarin-only users; 2) patients who switched to rivaroxaban from warfarin to warfarin-only users and 3) new rivaroxaban users to new dabigatran users. As noted above, anticoagulant users were initially matched by age, sex, enrolment date, and anticoagulant initiation date, for the purpose of defining an index date, and to collect covariate information at the time of drug initiation. To better compare the groups based on characteristics at the time of drug initiation, we then re-matched patients according to each outcome-specific HDPS. A greedy matching technique with a calliper of 0.25 of a standard-deviation of each HDPS was used to improve exchangeability [22]. Using the calliper, new rivaroxaban users were matched with up to 2 warfarin-only users, rivaroxaban switchers were matched with up to 4 warfarin-only users, and new rivaroxaban users were matched 1:1 with new dabigatran users.

Cox proportional hazards models were used to estimate the association between anticoagulant type and the time to each outcome (ischemic stroke, intracranial bleeding, MI, GI bleeding, and the 3 control outcomes of hip/pelvic fracture, breast/prostate cancer, and asthma). The start of follow-up began at the drug index date. Time to event was considered as the time to each outcome event, health plan disenrollment, or the end of study follow-up, whichever occurred first. For each outcome, Cox proportional hazards models were adjusted for age (continuous), sex, CHA_2_DS_2_-VASc (categorical), HDPS (continuous), and prevalent outcome at the index date. We performed two sensitivity analyses. First, we required patients to have been enrolled in the database for at least 180 days (instead of 90 days) before the first oral anticoagulant prescription. Second, we limited the analysis to those who had AF after January 1st, 2011, to minimize selective prescribing.

Effect modification by sex, age (<75, ≥75), CHA_2_DS_2_-VASc score (<2, ≥2), early vs. late outcomes (<90 days ≥90 days), and rivaroxaban dose strength were explored using stratified analysis. A *p*-value for interaction was obtained by adding a multiplicative term in the model (i.e. sex*drug). All statistical analyses were performed with SAS v 9.3 (SAS Inc., Cary, NC).

## Results

After exclusion criteria were applied, our study included 32,495 new rivaroxaban users matched to 45,496 warfarin-only users, 11,845 switchers to rivaroxaban matched to 43,904 warfarin-only users, and 16,957 new rivaroxaban users matched to 16,957 new dabigatran users. Numbers varied slightly across analyses due to endpoint-specific HDPS matching; therefore the total numbers listed in the tables are for the outcome of ischemic stroke. Characteristics of patients initiating rivaroxaban were comparable to their matched controls (Table 1). New rivaroxaban users were similar to new warfarin users, though slightly younger in age (mean age 69 vs. 71), and with a mean CHA_2_DS_2_-VASc score of 3.0 compared to 3.2 in warfarin-only users. Switchers to rivaroxaban from warfarin were comparable to their matched warfarin-only users, with a mean age of 71 and a mean CHA_2_DS_2_-VASc score of 4.0 vs. 3.9, respectively. New rivaroxaban users matched to new dabigatran users were similar, with the same mean age (67) and mean CHA_2_DS_2_-VASc score (2.6). Across comparison groups, the switchers (rivaroxaban switchers and their matched warfarin-only users) were older and had a higher prevalence of comorbidities (85% hypertensive, 35% diabetic, mean CHA_2_DS_2_-VASc score of 4.0) when compared to the new anticoagulant users. The new rivaroxaban and new dabigatran users tended to be younger (mean age 67) and had the fewest comorbidities among the comparison groups, with a mean CHA_2_DS_2_-VASc score of 2.6.

Overall, the characteristics of patients after the final matching (listed in Table 1) were similar to characteristics at the time of initial matching (listed in Additional file 1: Table S1). HDPS distributions for ischemic stroke by comparison group prior to HDPS matching are depicted in Additional file 1: Figure S1. The distributions are most similar between new rivaroxaban and new dabigatran users and least similar between new rivaroxaban users and new warfarin-only users. These distributions are prior to matching on HDPS; the extreme ends of each distribution were less likely to be included in analyses since it is less likely that there will be suitable matched patients.

### New rivaroxaban users vs. new warfarin users

**Fig. 1:**
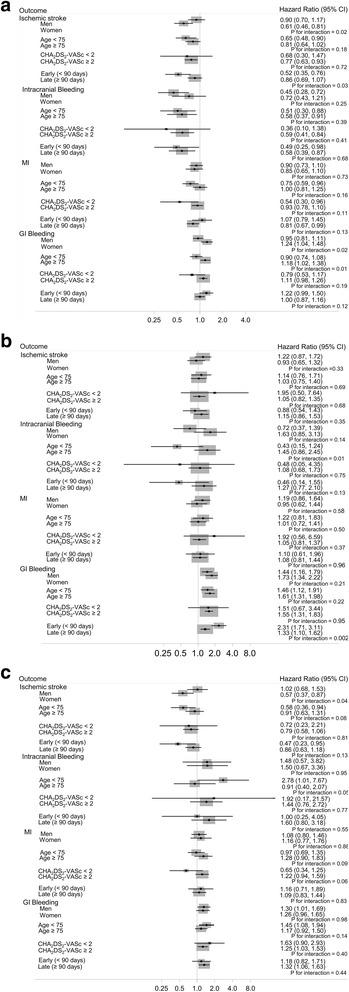
Adjusted hazard ratios (95% confidence intervals) of outcomes among anticoagulant users, stratified by subgroups, MarketScan, 2010–2014. **Panel a:** New rivaroxaban users vs. new warfarin users. **Panel b:** Patients who switched from warfarin to rivaroxaban vs. persistent warfarin users. **Panel c:** New rivaroxaban users vs. new dabigatran users

The hazard ratio (HR) and 95% confidence interval (CI) of each outcome for new rivaroxaban users compared to new warfarin users are reported in Table 2. During a mean follow-up of 12 months (median 10.5 months), new rivaroxaban users had a significantly lower rate of ischemic stroke and intracranial bleeds compared to warfarin users, HR (95% CI) = 0.75 (0.62–0.91) and 0.55 (0.39–0.78), respectively, in models adjusted for age, sex, CHA_2_DS_2_-VASc score, prevalent outcome and HDPS. Rates of MI and GI bleeding were comparable between the two groups. New users of rivaroxaban had a lower risk of the control outcome hip/pelvic fractures compared to warfarin initiators; however, there was no association between anticoagulant type and the other 2 control outcomes. In stratified analysis, the reduction in stroke risk among new rivaroxaban users vs. warfarin was larger in women compared to men (HR (95% CI) = 0.61 (0.46, 0.81) vs. 0.90 (0.70, 1.17); p for interaction = 0.02) and in the first 90 days after initiation compared to the subsequent time period (HR (95% CI) = 0.52 (0.35, 0.76) vs. 0.86 (0.69, 1.07); p for interaction = 0.03), (Fig. 1, panel A). Rivaroxaban initiation (vs. warfarin) was associated with increased risk of GI bleeding in women but not in men (HR: 1.24 vs. 0.95, respectively; p for interaction = 0.02) and in those age ≥ 75 compared to those <75 (HR: 1.18 vs. 0.90, respectively; p for interaction = 0.01).

**Table 2:** Adjusted hazard ratios (95% confidence intervals) of selected outcomes comparing new rivaroxaban users to new warfarin users for the treatment of non-valvular atrial fibrillation, MarketScan, 2010–2014

	Rivaroxaban User (*n* = 32,495)			Matched Warfarin User (*n* = 45,496)			Hazard Ratio (95% CI)^a^	*p*-value
Main outcomes	# Events	Person-years	IR (95% CI)	# Events	Person-years	IR (95% CI)		
Ischemic stroke	165	33,252	5.0 (4.3–5.8)	347	45,965	7.5 (6.8–8.4)	0.75 (0.62, 0.91)	0.003
Intracranial bleeding	46	33,309	1.4 (1.0–1.8)	124	45,958	2.7 (2.3–3.2)	0.55 (0.39, 0.78)	0.0008
Myocardial infarction	244	33,183	7.4 (6.5–8.3)	421	45,965	9.2 (8.3–10.1)	0.88 (0.75, 1.03)	0.11
Gastrointestinal bleeding	492	33,134	14.8 (13.6–16.2)	717	45,649	15.7 (14.6–16.9)	1.07 (0.95, 1.20)	0.29
Control outcomes								
Hip / pelvic fracture	194	33,214	5.8 (5.1–6.7)	408	45,866	8.9 (8.1–9.8)	0.83 (0.70, 0.99)	0.04
Breast / prostate cancer	272	33,236	8.2 (7.3–9.2)	419	45,941	9.1 (8.3–10.0)	0.92 (0.79, 1.08)	0.33
Asthma	443	33,157	13.4 (12.2–14.6)	606	45,769	13.2 (12.2–14.3)	0.99 (0.88, 1.13)	0.93

*IR* incidence rate, *CI* confidence interval

Incidence rate is per 1000 person-years

^a^Adjusted for age, sex, CHA_2_DS_2_-VASc score, prevalent outcome and high-dimensional propensity score

### Rivaroxaban switchers vs. persistent warfarin users

Patients who switched to rivaroxaban from warfarin had a significantly higher rate of GI bleeding compared to warfarin-only users, HR (95% CI) = 1.55 (1.32–1.83) (Table 3). There was no significant difference in the rate of ischemic stroke, intracranial bleeding, or MI. Rivaroxaban switchers had a lower rate of hip/pelvic fractures compared to warfarin users while there was no association for the other 2 control outcomes. In stratified analysis, the increased risk of GI bleeding associated with switching to rivaroxaban was higher in the first 90 days after switching to rivaroxaban compared to the risk greater than 90 days after switching, HR (95% CI) = 2.31 (1.71–3.11) vs. 1.33 (1.10–1.62), p for interaction = 0.002 (Fig. 1, panel B). Rivaroxaban was associated with reduced risk of intracranial bleeding in those age < 75 but not in individuals 75 and older (HR: 0.43 vs. 1.45, respectively; p for interaction = 0.01). However, this interaction should be interpreted with caution due to the small numbers in the <75 age group (4 events in rivaroxaban users and 33 in warfarin users). The association of rivaroxaban use, compared to warfarin, was significantly different between the anticoagulant-naïve and anticoagulant-experienced (switchers) groups for ischemic stroke [HR (95% CI) = 0.75 (0.62, 0.91) vs. 1.06 (0.83, 1.36); p for interaction 0.03] intracranial bleeding [HR (95% CI) = 0.55 (0.39, 0.78) vs. 1.04 (0.66, 1.65); p for interaction 0.03], and for GI bleeding [HR (95% CI) = 1.07 (0.95, 1.20) vs. 1.55 (1.32, 1.83); p for interaction <0.001].

**Table 3:** Adjusted hazard ratios (95% confidence intervals) of selected outcomes comparing patients who switched to rivaroxaban from warfarin to warfarin-only users for the treatment of non-valvular atrial fibrillation, MarketScan, 2010–2014

	Rivaroxaban Switcher (*n* = 11,845)			Matched Warfarin User (*n* = 43,904)			Hazard Ratio (95% CI) ^a^	*p*-value
Main outcomes	# Events	Person-years	IR (95% CI)	# Events	Person-years	IR (95% CI)		
Ischemic stroke	85	11,758	7.2 (5.8–8.9)	278	40,081	6.9 (6.2–7.8)	1.06 (0.83–1.36)	0.62
Intracranial bleeding	24	11,808	2.0 (1.3–3.0)	83	40,221	2.1 (1.7–2.5)	1.04 (0.66–1.65)	0.86
Myocardial infarction	77	11,776	6.5 (5.2–8.1)	252	40,283	6.3 (5.5–7.1)	1.08 (0.84–1.40)	0.55
Gastrointestinal bleeding	216	11,681	18.5 (16.1–21.1)	489	39,955	12.2 (11.2–13.4)	1.55 (1.32–1.83)	<0.0001
Control outcomes								
Hip / pelvic fracture	86	11,765	8.2 (6.6–9.9)	410	40,091	10.2 (9.3–11.3)	0.73 (0.58–0.92)	0.009
Breast / prostate cancer	107	11,749	9.1 (7.5–11.0)	297	40,164	7.4 (6.6–8.3)	1.21 (0.97–1.51)	0.10
Asthma	166	11,720	14.2 (12.1–16.4)	482	40,063	12.0 (11.0–13.1)	1.12 (0.94–1.34)	0.21

*IR* incidence rate, *CI* confidence interval

Incidence rate is per 1000 person-years

^a^Adjusted for age, sex, CHA_2_DS_2_-VASc score, prevalent outcome and high-dimensional propensity score

### New rivaroxaban users vs. new dabigatran users

Compared to new dabigatran users, those initiating rivaroxaban had a significantly higher risk of GI bleeding, HR (95% CI) = 1.28 (1.06–1.54) (Table 4). Rivaroxaban users had a non-significant lower risk of ischemic stroke compared to dabigatran users, HR (95% CI) = 0.77 (0.58–1.03), and this inverse association was present in women but not in men HR (95% CI) = 0.57 (0.37–0.87) vs. 1.02 (0.69–1.53), p for interaction = 0.04 (Fig. 1, panel C). There was no difference between rivaroxaban and dabigatran initiators in the risk of intracranial bleeds, MI, or any of the control outcomes, and no statistically significant interactions in the remaining stratified results.

**Table 4:** Adjusted hazard ratios (95% confidence intervals) of selected outcomes comparing new rivaroxaban users to new dabigatran users for the treatment of non-valvular atrial fibrillation, MarketScan, 2010–2014

	Rivaroxaban User (*n* = 16,957)			Matched Dabigatran User (*n* = 16,957)			Hazard Ratio (95% CI) ^a^	*p*-value
Main outcomes	# Events	Person-years	IR (95% CI)	# Events	Person-years	IR (95% CI)		
Ischemic stroke	82	21,721	3.8 (3.0–4.7)	107	21,723	4.9 (4.1–5.9)	0.77 (0.58–1.03)	0.08
Intracranial bleeding	26	21,787	1.2 (0.8–1.7)	17	21,774	0.8 (0.5–1.2)	1.47 (0.80–2.72)	0.22
Myocardial infarction	140	21,734	6.4 (5.4–7.6)	124	21,703	5.7 (4.8–6.8)	1.11 (0.87–1.41)	0.42
Gastrointestinal bleeding	255	21,633	11.8 (10.4–13.3)	198	21,641	9.1 (7.9–10.5)	1.28 (1.06–1.54)	0.01
Control outcomes								
Hip / pelvic fracture	101	21,770	4.6 (3.8–5.6)	116	21,706	5.3 (4.4–6.4)	0.88 (0.67–1.15)	0.34
Breast / prostate cancer	140	21,726	6.4 (5.4–7.6)	130	21,719	6.0 (5.0–7.1)	1.06 (0.84–1.35)	0.61
Asthma	245	21,645	11.3 (10.0–12.8)	203	21,629	9.4 (8.2–10.7)	1.18 (0.98–1.42)	0.09

*IR* incidence rate, *CI* confidence interval

Incidence rate is per 1000 person-years

^a^Adjusted for age, sex, CHA_2_DS_2_-VASc score, prevalent outcome and high-dimensional propensity score

### Associations by rivaroxaban dose

Results stratified by initial rivaroxaban dose are listed in Additional file 1: Tables S2-S4. Most patients were taking the 20 mg dose (74–80% for each comparison group), while only around 5% were taking the 10 mg dose, and the remaining percentage taking the 15 mg dose. Because of low numbers in the 10 mg group, and since this dose is not FDA-approved for NVAF treatment, we focus only on the 15 mg and 20 mg groups. Overall, the associations were similar between the 15 mg and 20 mg groups when compared to their matched warfarin or dabigatran comparison groups. The exception was that for new rivaroxaban users, the 15 mg group had a higher risk of MI, HR (95% CI) = 1.19 (0.93–1.52), and GI bleeding = 1.40 (1.17–1.65) than the 20 mg group, 0.77 (0.63–0.93) and 0.97 (0.84–1.11), respectively, when compared to the matched warfarin-only users.

### Sensitivity analyses

We performed a sensitivity analysis comparing new rivaroxaban users to new warfarin users, but restricted to patients with at least 180 days of enrolment before the first oral anticoagulation prescription. The results are listed in Additional file 1: Table S5, and indicate similar risks as our main results in Table 2, where we required 90 days of anticoagulation free enrolment. To account for selective prescribing based on FDA approval dates, we performed another sensitivity analysis in which restricted the analysis comparing new rivaroxaban users to new warfarin users to those with an enrolment date after January 1st, 2011. These results are listed in Additional file 1: Table S6 and are similar to our main results listed in Table 2.

## Discussion

In this retrospective administrative claims analysis of NVAF patients, we found that anticoagulant-naïve rivaroxaban initiators had lower risks of ischemic stroke and intracranial bleeding compared to new warfarin users. These benefits of rivaroxaban were not observed for patients switching from warfarin to rivaroxaban compared to persistent warfarin users. However, among patients switching to rivaroxaban, risk of GI bleeding was higher, especially in the first 90 days after switching. New rivaroxaban users and new dabigatran users had comparable rates of ischemic stroke and intracranial bleeding, but the former had a higher risk of GI bleeding.

Results from our analysis are mostly consistent with efficacy and safety results from clinical trial data and results from real-world studies where rivaroxaban was non-inferior or superior to warfarin for the prevention of stroke or systemic embolism [9, 13, 14, 34, 35]. In the ROCKET AF trial, which included 14,264 patients with NVAF randomized to 20 mg rivaroxaban once daily or dose-adjusted warfarin, rates of ischemic stroke, intracranial bleeding, and fatal bleeding were lower among individuals assigned to rivaroxaban [9]. In a sub-analysis of the ROCKET AF trial comparing efficacy and risks separately in vitamin K antagonist-naïve and vitamin K antagonist-experienced patients, rivaroxaban was associated with decreased risk of ischemic stroke and bleeding only in the former group [36]. Our results follow a similar pattern, indicating that switching from warfarin to rivaroxaban may not provide any additional benefit for stroke prevention. In addition, we observed that the effectiveness of rivaroxaban for stroke prevention was more accentuated in women than men, and in the first 90 days after initiation compared to more than 90 days after initiation. The benefit of rivaroxaban versus warfarin in the first 90 days after initiation may be explained by the need of warfarin users to adjust their dose while they stabilize in the therapeutic range. Patients may be at increased risk for cardioembolic events during that critical period [37].

In the head-to-head NOAC analysis, we found rivaroxaban users had a non-significant lower risk of ischemic stroke compared to dabigatran users, similar to what has recently been reported, [17] however our results indicate this inverse association was significantly stronger in women compared to men. This protective association of rivaroxaban (vs. dabigatran) with stroke risk in women was of similar magnitude to that seen for new female rivaroxaban users compared to warfarin users. Future analysis may explore whether rivaroxaban use is more beneficial in women for stroke prevention, compared to warfarin or dabigatran use.

An elevated risk of GI bleeding in rivaroxaban patients has been reported in several studies, [9, 12, 17, 35, 38] with an increased risk in those age > 75 [39]. Our results partially corroborate these findings. New rivaroxaban users did not have a significantly increased risk of GI bleeds compared to warfarin users, however, in stratified analysis, women and patients age ≥ 75 were at an increased risk of GI bleeding. New rivaroxaban users had a higher risk of GI bleeding when compared to dabigatran users. Patients who switched to rivaroxaban also had a higher risk of GI bleeding, especially in the first 90 days after switching anticoagulants. This increased risk of GI bleeding in switchers could be confounded by clinical factors that made patients switch from warfarin to a NOAC, such as an adverse reaction to or complication from using warfarin, or the patient’s inability to stabilize his/her warfarin dose. In our study, the switcher group was older and had more comorbidities compared to the new users, which are risk factors for GI bleeding. Further studies should examine patient characteristics in those who develop GI bleeding to identify in advance individuals most at risk of rivaroxaban-associated GI bleeding events, potentially using another oral anticoagulant in those patients.

Effectiveness and risks of rivaroxaban were similar by initial dose, except that new rivaroxaban users taking 15 mg had a higher risk of MI and GI bleeding compared to those prescribed the 20 mg dose. The 15 mg dose is indicated for patients with reduced kidney function, and that, along with other comorbidities associated with kidney disease, could be driving this association. The comparative effectiveness of NOACs in patients with reduced kidney function should be addressed in future research.

We observed an unexpected association between rivaroxaban users and a lower risk of hip/pelvic fracture (a control outcome), when compared to warfarin use. In addition to its established effect of impairing synthesis of vitamin K-associated clotting factors, warfarin is believed to inhibit the activation of bone proteins and, therefore, warfarin users could be at a higher risk of osteoporotic fractures compared to users of other oral anticoagulants [40]. However, observational studies have reported conflicting results on whether this association exists, [41, 42] and chance or residual confounding may be responsible for the finding in the present analysis. Given uncertainty regarding whether warfarin may increase fracture risk, hip/pelvic fracture is likely not an ideal ‘control’ outcome. Nonetheless, for transparency we chose to report these results as they were pre-specified in our analysis plan. Importantly, we did not see any association of rivaroxaban with the other two control outcomes -breast/prostate cancer and asthma – for which no interrelations with warfarin are hypothesized.

This study has several limitations which should be considered. First, unmeasured confounding is a known limitation in observational studies using administrative claims data. To account for confounding, we matched and adjusted for HDPS, which utilizes pre-defined variables and a wide range of empirically-identified confounders and has shown to be an effective approach for control of confounding [31]. However, in order to make causal interference, the two treatment groups need to be similar- that means that the final matched sample based on HDPS may not be representative from the entire treated population. Therefore, our results only apply to the matched population, which may be different from the entire treated population. In addition, we included control outcomes in our analysis, which we would not expect to be associated with anticoagulant use, providing indirect evidence of no residual uncontrolled confounding. Second, outcomes and covariates are ascertained from administrative data, which has known limitations. However, validated algorithms were utilized to ascertain events of interest and it is likely that any misclassification is non-differential. Although administrative data may fail to capture all comorbidities, the mean CHA_2_DS_2_-VASc score and the prevalence of comorbidities is similar to the comorbidities in patients included in the NOAC clinical trials and in NVAF patient registries. In addition, the outcomes of interest are serious enough to require medical care and, therefore, unlikely to be missed in this administrative database. Third, these results may not be generalizable to the entire population. Lastly, we did not confirm medication adherence. We report only initial prescription fill and dose, and did not include information on whether patients adhered to medication for the duration of the study period. There is no information on time in therapeutic range for the warfarin group. These patients may or may not be well-controlled. Persistence of NOACs is higher than warfarin, with rivaroxaban users having a persistence of 75–80% at 1 year [34, 43].

Despite these limitations, our study has numerous key strengths. This is a large, real-world population, with enough power to detect adverse outcomes over time. This study reports in-depth, stratified results (by sex, age, CHA_2_DS_2_-VASc score, and early vs. late outcomes) in patients who switched from warfarin to rivaroxaban, and also reports these stratified results for head-to-head comparisons between rivaroxaban and dabigatran users. In addition, we report associations for each outcome by initial rivaroxaban dose strength. These results provide information on the safety profile of rivaroxaban and may help clinicians make informed decisions when selecting an oral anticoagulant for thromboembolic prevention in NVAF patients.

## Conclusion

In conclusion, in this large real-world sample of NVAF patients, effectiveness and risks of rivaroxaban versus warfarin differed by prior anticoagulant status, while effectiveness of rivaroxaban versus dabigatran differed in GI bleeding risk. These results bolster prior clinical trials and observational studies, and provide more in-depth information on effectiveness and adverse events across patient subgroups, including those defined by prior use of warfarin.

## Additional file

Additional file 1: Supplemental Materials.
**Table S1.** Characteristics of atrial fibrillation patients by anticoagulant use prior to final matching based on high-dimensional propensity score, MarketScan, 2010–2014. **Table S2.** Adjusted hazard ratios (95% confidence intervals) of selected outcomes comparing new rivaroxaban users (categorized by initial dose) to new warfarin users for the treatment of non-valvular atrial fibrillation, MarketScan, 2010–2014. **Table S3.** Adjusted hazard ratios (95% confidence intervals) of selected outcomes comparing patients who switched to rivaroxaban (categorized by initial dose) from warfarin to warfarin-only users for the treatment of non-valvular atrial fibrillation, MarketScan, 2010–2014. **Table S4.** Adjusted hazard ratios (95% confidence intervals) comparing new rivaroxaban users (categorized by initial dose) to new dabigatran users for the treatment of non-valvular atrial fibrillation, MarketScan, 2010–2014. **Table S5.** Adjusted hazard ratios (95% confidence intervals) of selected outcomes comparing new rivaroxaban users to new warfarin users for the treatment of non-valvular atrial fibrillation, MarketScan, 2010–2014. Restricted to 36,623 patients with at least 180 days of enrolment before first oral anticoagulation prescription. **Table S6.** Adjusted hazard ratios (95% confidence intervals) of selected outcomes comparing new rivaroxaban users to new warfarin users for the treatment of non-valvular atrial fibrillation, MarketScan, 2010–2014. Restricted to 68,927 patients with an enrolment date later than January 1st, 2011. **Table S7.** ICD-9-CM codes for outcomes. **Table S8.** ICD-9-CM codes used to define comorbidities. **Figure S1.** High-dimensional propensity score distribution by oral anticoagulant status for the outcome of stroke. These are the distributions prior to final matching based on high-dimensional propensity score. (DOCX 356 kb)

## Abbreviations

AF: Atrial fibrillation
CI: Confidence interval
FDA: Food and Drug Administration
GI: Gastrointestinal
HDPS: High-dimensional propensity score
HR: Hazard ratio
ICB: Intracranial bleeding
ICD-9-CM: International Classification of Diseases, Ninth Revision, Clinical Modification
INR: International normalized ratio
MI: Myocardial infarction
NOACs: Non-vitamin K antagonist oral anticoagulants
NVAF: Non-valvular atrial fibrillation
PPV: Positive predicted value
US: United States
