# Supplementary material for: Comparative effectiveness of rivaroxaban versus warfarin or dabigatran for the treatment of patients with non-valvular atrial fibrillation
Source: BMC Cardiovasc Disord. 2017 Sep 6;17:238. doi: 10.1186/s12872-017-0672-5 (PMC5585896; doi:10.1186/s12872-017-0672-5)
Supplement: Additional file 1: Supplemental Materials. — Table S1. Characteristics of atrial fibrillation patients by anticoagulant use prior to final matching based on high-dimensional propensity score, MarketScan, 2010–2014. Table S2. Adjusted hazard ratios (95% confidence intervals) of selected outcomes comparing new rivaroxaban users (categorized by initial dose) to new warfarin users for the treatment of non-valvular atrial fibrillation, MarketScan, 2010–2014. Table S3. Adjusted hazard ratios (95% confidence intervals) of selected outcomes comparing patients who switched to rivaroxaban (categorized by initial dose) from warfarin to warfarin-only users for the treatment of non-valvular atrial fibrillation, MarketScan, 2010–2014. Table S4. Adjusted hazard ratios (95% confidence intervals) comparing new rivaroxaban users (categorized by initial dose) to new dabigatran users for the treatment of non-valvular atrial fibrillation, MarketScan, 2010–2014. Table S5. Adjusted hazard ratios (95% confidence intervals) of selected outcomes comparing new rivaroxaban users to new warfarin users for the treatment of non-valvular atrial fibrillation, MarketScan, 2010–2014. Restricted to 36,623 patients with at least 180 days of enrolment before first oral anticoagulation prescription. Table S6. Adjusted hazard ratios (95% confidence intervals) of selected outcomes comparing new rivaroxaban users to new warfarin users for the treatment of non-valvular atrial fibrillation, MarketScan, 2010–2014. Restricted to 68,927 patients with an enrolment date later than January 1st, 2011. Table S7. ICD-9-CM codes for outcomes. Table S8. ICD-9-CM codes used to define comorbidities. Figure S1. High-dimensional propensity score distribution by oral anticoagulant status for the outcome of stroke. These are the distributions prior to final matching based on high-dimensional propensity score. (DOCX 356 kb) [file 12872_2017_672_MOESM1_ESM.docx]

**SUPPLEMENTAL MATERIAL**

Norby et al. *Comparative Effectiveness of Rivaroxaban Versus Warfarin or Dabigatran for the Treatment of Patients with Non-valvular Atrial Fibrillation*

**Supplemental Tables**

Table 1. Characteristics of atrial fibrillation patients by anticoagulant use prior to final matching based on high-dimensional propensity score, MarketScan, 2010-2014

|  | New rivaroxaban (n=39,011) | New warfarin (n=83,981) | New dabigatran (n=18,109) | Switchers from warfarin to rivaroxaban (n=11,860) |
| --- | --- | --- | --- | --- |
| Age, years | 68.1 ± 12.3 | 70.8 ± 12.4 | 67.2 ± 12.0 | 71.2 ± 12.1 |
| Age ≥ 75 years | 33.1 | 42.6 | 29.8 | 44.3 |
| Female, % | 37.7 | 39.4 | 34.3 | 39.3 |
| Comorbidities, % |  |  |  |  |
| Hypertension | 68.3 | 66.4 | 59.1 | 84.8 |
| Diabetes | 25.6 | 30.3 | 23.7 | 35.3 |
| Myocardial infarction | 6.8 | 9.8 | 5.4 | 11.2 |
| Heart failure | 22.0 | 30.9 | 19.4 | 38.7 |
| Ischemic stroke/TIA | 14.8 | 19.5 | 12.3 | 29.1 |
| Hemorrhagic stroke | 0.5 | 1.1 | 0.4 | 1.7 |
| PAD | 11.4 | 17.2 | 8.5 | 23.7 |
| Dementia | 1.0 | 1.8 | 0.7 | 2.9 |
| Renal Disease | 6.9 | 14.8 | 5.1 | 14.1 |
| Chronic pulmonary disease | 21.0 | 25.0 | 17.1 | 34.6 |
| Liver disease | 3.6 | 4.5 | 2.9 | 7.0 |
| Malignancy | 10.7 | 12.4 | 7.9 | 17.1 |
| Depression | 7.3 | 8.9 | 5.7 | 13.7 |
| Hematological disorders | 7.2 | 14.8 | 5.1 | 22.5 |
| Metastatic cancer | 1.5 | 2.2 | 0.9 | 2.7 |
| Alcohol abuse | 0.4 | 0.4 | 0.3 | 0.6 |
| GI bleed | 4.3 | 6.5 | 3.2 | 12.1 |
| Other bleed | 2.3 | 4.3 | 1.5 | 8.3 |
| CHA_2_DS_2_-VASC score | 2.9 ± 1.9 | 3.4 ± 2.0 | 2.6 ± 1.8 | 4.0 ± 2.1 |
| CHA_2_DS_2_-VASC score ≥ 2 | 72.5 | 82.2 | 68.6 | 87.4 |
| Prior procedures, % |  |  |  |  |
| Cardiac | 57.5 | 63.4 | 50.6 | 81.2 |
| Vascular | 4.0 | 9.7 | 2.7 | 9.8 |
| Gastrointestinal | 21.6 | 23.9 | 16.8 | 42.4 |
| Neurological | 13.5 | 12.5 | 8.4 | 23.8 |
| Medications, % |  |  |  |  |
| Digoxin | 10.7 | 15.3 | 11.3 | 23.0 |
| Clopidogrel | 9.3 | 9.5 | 7.2 | 11.0 |
| Antiplatelets | 1.7 | 1.7 | 1.2 | 2.1 |
| Angiotensin-converting enzyme inhibitors | 29.7 | 33.0 | 28.4 | 41.7 |
| Angiotensin receptor blockers | 20.9 | 19.2 | 17.6 | 26.5 |
| Beta-blockers | 65.1 | 64.0 | 60.9 | 77.3 |
| Calcium channel blockers | 36.5 | 35.2 | 32.9 | 46.5 |
| Anti-arrhythmias | 22.4 | 18.8 | 21.6 | 32.9 |
| Statins | 46.4 | 50.9 | 42.5 | 62.2 |
| Diabetes medications | 19.1 | 22.9 | 19.6 | 25.4 |

Values correspond to mean ± standard deviation or percentage

Table 2. Adjusted hazard ratios (95% confidence intervals) of selected outcomes comparing new rivaroxaban users (categorized by initial dose) to new warfarin users for the treatment of non-valvular atrial fibrillation, MarketScan, 2010-2014

|  | Rivaroxaban 10mg  (n=2,010) | Rivaroxaban 15mg  (n=5,922) | Rivaroxaban 20mg (n=24,563) | Matched Warfarin User (n=45,496) | Rivaroxaban 10mg Hazard Ratio  (95% CI) | Rivaroxaban 15mg  Hazard Ratio  (95% CI) | Rivaroxaban 20mg Hazard Ratio  (95% CI) |
| --- | --- | --- | --- | --- | --- | --- | --- |
| Main outcomes | # Events | # Events | # Events | # Events |  |  |  |
| Ischemic stroke | 14 | 47 | 104 | 347 | 0.81 (0.47, 1.38) | 0.74 (0.54, 1.01) | 0.75 (0.60, 0.94) |
| Intracranial bleeding | 1 | 14 | 31 | 124 | 0.15 (0.02, 1.16) | 0.66 (0.38, 1.16) | 0.56 (0.37, 0.84) |
| Myocardial infarction | 16 | 80 | 148 | 421 | 0.78 (0.47, 1.28) | 1.19 (0.93, 1.52) | 0.77 (0.63, 0.93) |
| Gastrointestinal bleeding | 27 | 166 | 299 | 717 | 0.76 (0.52, 1.12) | 1.40 (1.17, 1.65) | 0.97 (0.84, 1.11) |
| Adjusted for age, sex, CHA_2_DS_2_-VASc score, prevalent outcome and high-dimensional propensity score | | | | | | | |

Table 3. Adjusted hazard ratios (95% confidence intervals) of selected outcomes comparing patients who switched to rivaroxaban (categorized by initial dose) from warfarin to warfarin-only users for the treatment of non-valvular atrial fibrillation, MarketScan, 2010-2014

|  | Rivaroxaban 10mg  (n=639) | Rivaroxaban 15mg  (n=2,481) | Rivaroxaban 20mg (n=8,725) | Matched Warfarin User (n=43,904) | Rivaroxaban 10mg Hazard Ratio  (95% CI) | Rivaroxaban 15mg  Hazard Ratio  (95% CI) | Rivaroxaban 20mg Hazard Ratio  (95% CI) |
| --- | --- | --- | --- | --- | --- | --- | --- |
| Main outcomes | # Events | # Events | # Events | # Events |  |  |  |
| Ischemic stroke | 8 | 22 | 55 | 278 | 1.65 (0.82, 3.34) | 0.92 (0.59, 1.42) | 1.08 (0.80, 1.45) |
| Intracranial bleeding | 1 | 11 | 12 | 83 | 0.69 (0.10, 4.99) | 1.66 (0.87, 3.15) | 0.80 (0.43, 1.48) |
| Myocardial infarction | 6 | 25 | 46 | 252 | 1.34 (0.60, 3.02) | 1.19 (0.79, 1.81) | 1.01 (0.73, 1.38) |
| Gastrointestinal bleeding | 6 | 65 | 145 | 489 | 0.66 (0.29, 1.47) | 1.66 (1.27, 2.15) | 1.60 (1.32, 1.93) |
| Adjusted for age, sex, CHA_2_DS_2_-VASc score, prevalent outcome and high-dimensional propensity score | | | | | | | |

Table 4. Adjusted hazard ratios (95% confidence intervals) comparing new rivaroxaban users (categorized by initial dose) to new dabigatran users for the treatment of non-valvular atrial fibrillation, MarketScan, 2010-2014

|  | Rivaroxaban 10mg  (n=818) | Rivaroxaban 15mg  (n=2,572) | Rivaroxaban 20mg (n=13,567) | Matched Dabigatran User (n=16,957) | Rivaroxaban 10mg Hazard Ratio  (95% CI) | Rivaroxaban 15mg  Hazard Ratio  (95% CI) | Rivaroxaban 20mg Hazard Ratio  (95% CI) |
| --- | --- | --- | --- | --- | --- | --- | --- |
| Main outcomes | # Events | # Events | # Events | # Events |  |  |  |
| Ischemic stroke | 8 | 22 | 52 | 107 | 1.14 (0.55, 2.36) | 0.72 (0.45, 1.15) | 0.76 (0.54, 1.06) |
| Intracranial bleeding | 0 | 6 | 20 | 17 | N/A | 1.35 (0.52, 3.50) | 1.64 (0.85, 3.16) |
| Myocardial infarction | 11 | 44 | 85 | 124 | 1.38 (0.74, 2.57) | 1.54 (1.08, 2.21) | 0.95 (0.72, 1.25) |
| Gastrointestinal bleeding | 11 | 74 | 170 | 198 | 0.82 (0.45, 1.52) | 1.35 (1.02, 1.77) | 1.30 (1.05, 1.60) |
| Adjusted for age, sex, CHA_2_DS_2_-VASc score, prevalent outcome and high-dimensional propensity score | | | | | | | |

Table 5. Adjusted hazard ratios (95% confidence intervals) of selected outcomes comparing new rivaroxaban users to new warfarin users for the treatment of non-valvular atrial fibrillation, MarketScan, 2010-2014. Restricted to 36,623 patients with at least 180 days of enrolment before first oral anticoagulation prescription.

|  | Hazard Ratio (95% Confidence Interval) | p-value |  |
| --- | --- | --- | --- |
| Main outcomes |  |  |  |
| Ischemic stroke | 0.77 (0.60, 0.98) | 0.04 |  |
| Intracranial bleeding | 0.52 (0.32, 0.84) | 0.008 |  |
| Myocardial infarction | 0.80 (0.63, 1.03) | 0.08 |  |
| Gastrointestinal bleeding | 1.13 (0.95, 1.35) | 0.18 |  |
| Control outcomes |  |  |  |
| Hip / pelvic fracture | 0.92 (0.71, 1.18) | 0.51 |  |
| Breast / prostate cancer | 0.87 (0.70, 1.08) | 0.21 |  |
| Asthma | 1.04 (0.86, 1.25) | 0.69 |  |
| Adjusted for age, sex, CHA_2_DS_2_-VASc score, prevalent outcome and high-dimensional propensity score | | | |

Table 6. Adjusted hazard ratios (95% confidence intervals) of selected outcomes comparing new rivaroxaban users to new warfarin users for the treatment of non-valvular atrial fibrillation, MarketScan, 2010-2014. Restricted to 68,927 patients with an enrolment date later than January 1^st^, 2011.

|  | Hazard Ratio (95% Confidence Interval) | p-value |  |
| --- | --- | --- | --- |
| Main outcomes |  |  |  |
| Ischemic stroke | 0.73 (0.60, 0.91) | 0.004 |  |
| Intracranial bleeding | 0.61 (0.43, 0.89) | 0.01 |  |
| Myocardial infarction | 0.88 (0.74, 1.04) | 0.14 |  |
| Gastrointestinal bleeding | 1.05 (0.93, 1.21) | 0.38 |  |
| Control outcomes |  |  |  |
| Hip / pelvic fracture | 0.80 (0.66, 0.97) | 0.02 |  |
| Breast / prostate cancer | 0.96 (0.81, 1.14) | 0.65 |  |
| Asthma | 0.99 (0.86, 1.14) | 0.89 |  |
| Adjusted for age, sex, CHA_2_DS_2_-VASc score, prevalent outcome and high-dimensional propensity score | | | |

**Supplemental Figures**

Figure 1. High-dimensional propensity score distribution by oral anticoagulant status for the outcome of stroke. These are the distributions prior to final matching based on high-dimensional propensity score.

Panel A. High-dimensional propensity score distribution for the outcome of stroke, stratified by warfarin and rivaroxaban users

Warfarin mean =0.22

Rivaroxaban mean =0.52

0

2

4

6

8

10

12

14

Percent

0

0.045

0.090

0.135

0.180

0.225

0.270

0.315

0.360

0.405

0.450

0.495

0.540

0.585

0.630

0.675

0.720

0.765

0.810

0.855

0.900

0.945

0.990

0

2

4

6

8

10

12

14

Percent

Rivaroxaban

Estimated Probability

Warfarin

Panel B. High-dimensional propensity score distribution for the outcome of stroke, stratified by warfarin and switchers from warfarin to rivaroxaban

Rivaroxaban switcher mean =0.21

Warfarin mean =0.16

0

1

2

3

4

5

6

7

8

Percent

0

0.048

0.096

0.144

0.192

0.240

0.288

0.336

0.384

0.432

0.480

0.528

0.576

0.624

0.672

0.720

0

1

2

3

4

5

6

7

8

Percent

Estimated Probability

Rivaroxaban

Warfarin

Panel C. High-dimensional propensity score distribution for the outcome of stroke, stratified by dabigatran and rivaroxaban users

Dabigatran mean =0.48

Rivaroxaban mean =0.52

0

5

10

15

20

Percent

0.075

0.125

0.175

0.225

0.275

0.325

0.375

0.425

0.475

0.525

0.575

0.625

0.675

0.725

0.775

0.825

0.875

0.925

0.975

Percent

Estimated Probability

Rivaroxaban

Dabigatran

10

15

20

5

0

Table 7. ICD-9-CM codes for outcomes

| **Condition** | **ICD-9-CM codes** |
| --- | --- |
| Ischemic stroke | 434, 436 |
| Intracranial bleeding | 430, 431 |
| Myocardial infarction | 410, 412 |
| Gastrointestinal bleeding | 455.2, 455.5, 455.8, 456.0, 456.20, 530.7, 530.82, 531.0, 531.2, 531.4, 531.6, 532.0, 532.2, 532.4, 532.6, 533.0, 533.2, 533.4, 533.6, 534.0, 534.2, 534.4, 534.6, 535.01, 535.11, 535.21, 535.31, 535.41, 535.51, 535.61, 537.83, 562.02, 562.03, 562.12, 562.13, 568.81, 569.3, 569.85, 578.0, 578.1, 578.9 |
| Hip fracture | 733.14, 733.15, 733.96, 733.97, 733.98, 808.0, 808.1 808.2 808.3 808.41, 808.42, 808.43, 808.44, 808.49, 808.51, 808.52, 808.53, 808.54, 808.59, 808.8, 808.9, 820.00, 820.01, 820.02, 820.03, 820.09, 820.10, 820.11, 820.12, 820.13, 820.19, 820.20, 820.21, 820.22, 820.30, 820.31, 820.32, 820.8, 820.9 |
| Breast / prostate cancer | 174.x, 175.0, 175.9, 233.0, V103  185, 233.4, V1046 |
| Asthma | 493.00, 493.01, 493.02, 493.10, 493.11, 493.12, 493.20, 493.21, 493.22, 493.81, 493.82, 493.90, 493.91, 493.92 |

Table 8. ICD-9-CM codes used to define comorbidities

| Condition | ICD-9-CM codes |
| --- | --- |
| Alcoholism | 265.2, 291.1, 291.2, 291.3, 291.5, 291.6, 291.7, 291.8, 291.9, 303.0, 303.9, 305.0, 357.5, 425.5, 535.3, 571.0, 571.1, 571.2, 571.3, 980, V11.3 |
| Hematological disorders (Coagulopathy, anemia) | 280, 281, 282, 283, 284, 285, 286, 287.1, 287.3, 287.4, 287.5 |
| Cancer | 140-172, 174-195, 200-208, 238.6 |
| Chronic pulmonary disease | 416.8, 416.9, 490-505, 506.4 508.1 508.8 |
| Dementia | 290.x, 294.1, 331.2 |
| Depression | 296.2, 296.3, 296.5, 300.4, 309.x, 311.x |
| Diabetes | 250 |
| Heart failure | 398.91, 402.01, 402.11, 402.91, 404.01, 404.03, 404.11, 404.13, 404.91, 404.93, 425.4, 425.9, 428 |
| Hypertension | 401, 402, 403, 404, 405 |
| Kidney disease | 403.01, 403.11, 403.91, 404.02, 404.03, 404.12, 404.13, 404.92, 404.93, 582, 583.0, 583.1, 583.2, 583.3, 583.4, 583.5, 583.6, 583.7, 585, 586, 588.0, V42.0, V45.1, V56 |
| Liver disease | 070.22, 070.23, 070.32, 070.33, 070.44, 070.54, 070.6, 070.9, 456.0, 456.1, 456.2, 570, 571, 572.2, 572.3, 572.4, 572.5, 572.6, 572.7, 572.8, 573.3, 573.4, 573.8, 573.9, V42.7 |
| Other bleeding | 423.0, 459.0, 568.81, 593.81, 599.7, 623.8, 626.6, 719.1, 784.7, 784.8, 786.3 |
| Peripheral artery disease | 440.0, 440.2, 440.9, 441, 443.9 |
| Procedure codes |  |
| Cardiac | 35, 36, 37 |
| Vascular | 38, 39 |
| Gastrointestinal | 42-54 |
| Neurological | 01-05 |
| Medications | Therapeutic detail or class code (as used in MarketScan databases) |
| Digoxin | 2404080040 |
| Clopidogrel | 2060010008 |
| Antiplatelets | 2060010005 2060010007 2060010009 2060010010 2060010020 2060010030 2060010045 2060010050 2060010060 |
| Angiotensin-converting enzyme inhibitors | 47 |
| Angiotensin receptor blockers | 2404010018 2404010020 2404010030 2404010032 2404010037 2404010040 2404010053 2404010055 2404010090 |
| Beta-blockers | 51 |
| Calcium channel blockers | 52 |
| Anti-arrhythmias | 49 |
| Statins | 2406010005 2406010008 2406010047 2406010070 2406010078 2406010080 2406010095 2406010099 2406010100 |
| Diabetes medications | 6820080000 6820080010 6820080020 6820080030 6820080050 6820200010 6820200020 6820200025 6820200030 6820200035 6820200070 6820200075  6820920001 6820920002 6820920003 6820920005 6820920007 6820920008 6820920010 6820920012 6820920015 6820920016 6820920017 6820920018 6820920019  6820920020 6820920021 6820920022 6820920023 6820920024 6820920025 6820920026 6820920030 6820920040 6820920043 6820920050 6820920058 6820920060  6820920070 6820920080 |
